# Citizen Science in the Natural Sciences

**DOI:** 10.1007/978-3-030-58278-4_5

**Published:** 2020-08-29

**Authors:** Didone Frigerio, Anett Richter, Esra Per, Baiba Pruse, Katrin Vohland

**Affiliations:** 1 Museum für Naturkunde Berlin – Leibniz, Institute for Evolution and Biodiversity Science (MfN), Berlin, Germany; 2 Faculty of Science, Leiden University, Leiden, The Netherlands; 3 Earthwatch Europe, Oxford, UK; 4 Faculty of Geo-Information Science and Earth Observation (ITC), University of Twente, Enschede, The Netherlands; 5 OpenSystems, Departament de Física de la Matèria Condensada, Universitat de Barcelona, Barcelona, Spain; 6 Department of Applied Information Technology, University of Gothenburg, Gothenburg, Sweden; 7 Department of Bioscience Engineering, University of Antwerp, Antwerp, Belgium; 8 Museum für Naturkunde Berlin – Leibniz, Institute for Evolution and Biodiversity Science (MfN), Berlin, Germany; 9 Konrad Lorenz Research Center for Behaviour and Cognition, University of Vienna, Vienna, Austria; 10 Department of Behavioural and Cognitive Biology, University of Vienna, Vienna, Austria; 11 Thünen Institute of Biodiversity, Brunswick, Germany; 12 Department of Biology, Faculty of Science, Gazi University, Ankara, Turkey; 13 Institute for Environmental Solutions and University of Latvia, Riga, Latvia; 14 Museum für Naturkunde Berlin – Leibniz Institute for Evolution and Biodiversity Science (MfN), Berlin, Germany; 15 Natural History Museum (NHM), Vienna, Austria

**Keywords:** Data-driven research, Empirical research, Theoretical research, Biodiversity monitoring, DIY biology

## Abstract

The natural sciences include the life and physical sciences and study nature through observing and understanding phenomena, testing hypotheses, and performing experiments. Key principles such as reliability, validity, objectivity, and predictability are achieved through transparent assumptions, methods, data, and interpretations as well as multidisciplinarity.

In this chapter we present insights into the genesis of citizen science in the natural sciences and reflect on the intellectual history of the natural sciences in relation to citizen science today. Further, we consider the current scientific approaches and achievements of natural science projects, which are applying citizen science to address empirical and/or theoretical research, focusing on monitoring programmes. Presenting examples and case studies, we focus on the key characteristics of the scientific inquiries being investigated in the natural sciences through citizen science. Finally, we discuss the consequences of engagement in scientific processes in relation to the future of natural scientists in a complex world.

## Introduction

The *natural sciences* combine the *life sciences*, which involve the study of life and organisms such as microorganisms, plants, and animals including human beings, and the *physical sciences*, which are focused on non-living systems such as celestial objects and the structure and composition of matters and substances. The natural sciences are grounded in observing and understanding phenomena, testing hypotheses, and performing experiments. *Inquiry-based research* is performed across spatial and temporal scales with the application of standardised methods and protocols. The main driver in the natural sciences can be expressed by Goethe’s Faust: ‘So that I may perceive whatever holds / The world together in its inmost folds’ (Goethe 1986). Information about the natural world is described in measurable units. Key principles such as reliability, repeatability, objectivity, and predictability ensure validity for scientific advances and are often achieved through multidisciplinary approaches. Both the life and physical sciences include several basic and applied scientific fields. Zoology, botany, genetics, neuroscience, and theoretical biology are examples of *basic research fields* in the life sciences, whereas environmental sciences and conservation biology are *applied research fields*. In turn, earth science, chemistry, physics, and astronomy are regarded as basic research fields in the physical sciences, whereas astrophysics, digital electronics, and nanotechnology are examples of applied research fields.

As a research format, *citizen science* has evolved over decades – generating knowledge, fostering scientific literacy, and enhancing learning through engagement in all scientific disciplines (Kullenberg and Kasperowski 2016). In this respect, the natural sciences offer a wide application for citizen science approaches across a range of disciplines (Follett and Strezov 2015). In the physical sciences, Galaxy Zoo is a well-known citizen science project, where the public was invited to visually inspect and classify nearly one million galaxies via the Internet. The aim of the study was to first distinguish between the two main morphological classes of massive systems in order to understand the formation and subsequent evolution of galaxies. The project achieved more than 40 million individual classifications made by hundreds of thousands of participants (Lintott et al. 2008). Sørensen et al. (2016) also launched Quantum Moves, an online project gamifying optimisation problems in quantum physics. The physicists showed that human players were able to find solutions to difficult problems associated with quantum computing. Furthermore, Barr et al. (2017) demonstrated that *non-expert volunteers* can identify the decay of long-lived particles with an efficiency and fake rate comparable to that of ATLAS algorithms, a machine learning-based analysis process.

Several examples of the successful application of citizen science can also be found in the life sciences. The project EteRNA was among the first Internet-scale citizen science games scored by high-throughput experiments. A community of 37,000 non-experts leveraged continuous remote laboratory feedback to learn new design rules that substantially improved the experimental accuracy of RNA structure design (Lee et al. 2014). Similarly, Phylo involved volunteers in investigating the multiple sequence alignment problem, used to reveal conserved DNA sequences across species (Singh et al. 2017). However, *biodiversity monitoring* projects are among the most common citizen science projects in the life sciences. For example, the North American Bird Phenology Program’s Migration Observer Cards project was among the earliest citizen science activities and has contributed vital data to ornithology (Irwin 1995; see also Box 5.1). Over recent decades, communities of non-expert volunteers have been involved in numerous projects, for example, in monitoring streams and benthic macro-invertebrates (Fore et al. 2001); in mapping the distribution of the wintering areas of monarch butterflies (Howard et al. 2010); in investigating the ecology of an invasive population of Red-vented Bulbuls (Brooks 2013); and in recording damage caused by leaf-mining moths to horse chestnuts (Pocock and Evans 2014).

In this chapter we explore and present insights into the genesis of citizen science in the natural sciences and reflect on the intellectual history of the natural sciences in relation to citizen science today. Specifically, we draw a line from the amateur scientists of the past working in isolated knowledge domains to the collaboration-based scientific investigations of the future. We start with the question ‘How are key characteristics of the natural sciences applied by contemporary citizen science?’ moving on to ‘What processes of scientific inquiry are investigated through citizen science?’ Finally, we explore the more theoretical question ‘What are the consequences of engagement in scientific processes?’, in respect to the future of natural scientists in a complex world.

Bringing together existing research, it becomes evident that while citizen science is well established in the natural sciences, no thematic boundaries seem to exist to integrate and make use of its manifold potential. As one of the major aims of this book is to give an overview about the current discussion, understanding, and relevance of citizen science in different scientific fields including humanities (Heinisch et al., Chap. 10.1007/978-3-030-58278-4_6, this volume) and the social sciences (Albert et al., Chap. 10.1007/978-3-030-58278-4_7, this volume), we begin our reflection about citizen science in the natural sciences by paying tribute to citizen scientist pioneers who inspired many of today’s citizen science enthusiasts. We introduce amateur scientists from the past as role models to learn about the key principles of citizen science in the natural sciences. We provide insights into the research approaches within projects and programmes and highlight scientific achievements as well as societal outcomes from citizen science in the natural sciences. For instance, selected case studies on biodiversity monitoring are presented to showcase practical aspects of citizen science in the natural sciences. The chapter closes with some remarks on the future.

## History

The history of the natural sciences and citizen science is closely related. One of the oldest examples which can be termed citizen science is the observation of cherry tree flowering in Kyoto, Japan (Aono and Kazui 2007). Merchants, politicians, monks, and others all noted the start of the cherry bloom in their diaries, the first entry found for which was from 801 AD.

When citizen science is presented to newcomers, historical stories, people, and places that have shaped the understanding of today’s natural world are often referred to.^1^ Explorers and advocates for the natural sciences such as Alexander von Humboldt, Ferdinand Müller, and Maria Sibylla Merian are showcased to highlight how enthusiasm and an inherent curiosity for understanding the natural world have influenced them. They travelled around the world into unknown and unexplored areas, collected information and data in remote and untouched places, archived species and objects in boxes and books to be shipped around the world, and shared new knowledge. Many amateur scientists from the past have shaped and grounded the natural sciences and are representative of the core of citizen science – seeking understanding and gaining new knowledge, sharing and caring for the sustainability of findings and data, and enabling members of society to become scientifically literate citizens.

When German-born Ferdinand Müller arrived in Australia in the mid-eighteenth century, the science of Australian botany was born. The amateur botanist devoted his life to the inventory of the iconic flora of the Terra Australis Incognita. He revolutionised data collection by engaging local communities and establishing groups of collectors that he recruited through newspaper articles and word-of-mouth recommendations. Over 1300 people, including Indigenous Australians, women, and children, supported Müller’s scientific mission and contributed to Australian botany (Finkel 2018).

In Europe, 200 years before Müller’s community engagement in scientific discovery, Maria Sibylla Merian was born in Frankfurt, Germany. A young Merian pursued her inquisitiveness of the world of insects and observed, described, and painted insect development, today known as metamorphosis. Later in life, she travelled as an amateur entomologist, with no formal education, around the world to investigate relationships between insects and host plants and developed, through her research, the foundation of modern entomology. Much of today’s knowledge, for example, about the distinctions between butterflies and moths and the ecological requirements for the survival of butterflies, dates to Merian’s early findings. Her knowledge and discoveries were published in several books in German (not in Latin, as was common at that time) and were, therefore, accessible to other non-scientists. However, she received less scientific recognition than her academic peers. Decades later, Merian is a widely recognised and respected figure in science.

In 2019, many research institutions across the world celebrated the 250th anniversary of Alexander von Humboldt and paid tribute to the universal scholar who developed and linked the knowledge of disciplines within the natural sciences, ranging from astronomy to zoology. Humboldt was an outstanding scholar who embodied the concept of life-long learning and widely communicated about science, writing thousands of letters to both his peers and policy-makers. His open mind motivated him to formulate and include new theses in his thinking about the natural world. Humboldt gained novel insights into global relationships such as the effects of human activities on climate change and the science of biogeography.

These three historical examples showcase that some of today’s key principles of citizen science (Robinson et al. 2018) are not new. The genesis of new scientific knowledge often starts with an observation of a phenomenon, and, as Charles Darwin pointed out, ‘It is well to remember that Naturalists value observations far more than reasoning’ (Darwin 1887). Observations are necessary to formulate research questions and testable hypotheses, ideally generating theories and leading to new questions requiring further observations. Both Humboldt and Merian applied this type of process when approaching the natural world. The explorers, universal scholars, and amateur scientists outlined reflect the beginning of the so-called professionalisation of science, when ‘doing science’ became a profession (for more details see Haklay et al., Chap. 10.1007/978-3-030-58278-4_2, this volume). Long before a distinction between amateurs and professional scientists was made, people with various backgrounds shared an interest to make sense of the world around them through community building and partnerships with members from various knowledge domains. Much of the understanding of the natural world was and is achieved through the development of standardised methods in the natural sciences.

## Research Approaches

Generally, the natural sciences can be defined as applying to ‘subject matter based on the philosophy of naturalism’ (Ledoux 2002), where natural events are investigated using scientific methods. In this chapter, we consider research in the domain of natural sciences to be grouped according to two major methodological domains – empirical and theoretical research. *Empirical research* can be distinguished between (1) *observations*, that is, the collection of data about objects in the natural world, and (2) *experiments*, that is, the collection of information and relations using variables and measurements that allow analysis of cause and effect relationships. Observations include the recording of patterns and processes occurring in, and being representative of, the natural world alongside various spatial and temporal dimensions, ranging from local to global phenomena and from short- to long-term observations. The observations are achieved using *senses* or *sensors*. Technical devices such as microscopes and scanners are used to further enhance the seeing, hearing, smelling, and feeling of objects in the natural world. Within experiments, the empirical approach is to collect evidence that confirms or rejects a hypothesis or assumption formulated prior to the collection of the evidence. This process allows the analysis of causal and/or correlative relationships. In contrast to the empirical domain that is data driven and focuses on testing and validating hypotheses, the theoretical domain is conceptually grounded and focuses on the collection of concepts and theses to explore and explain the natural world. For this, ideas are theorised, abstracted, and synthesised to find ways to define how the natural world and its environment interact (Lederman 2007). A pan-European survey showed that the majority of citizen projects involve performing observations and collecting data, rather than doing experiments or brainstorming on possible research questions (Hecker et al. 2018).

Biodiversity monitoring especially profits from a citizen science approach as working with citizen scientists in monitoring biodiversity networks increases the amount of data (and therefore their reliability) and expands the temporal and spatial scales of the investigation (Chandler et al. 2017a). As over 80% of biodiversity data in Europe is recorded by citizen scientists (Schmeller et al. 2009), it is not surprising that the spatial and temporal coverage of the assessment of biodiversity depends to a large degree on volunteers’ availability and ability to travel to areas of interest.

Such monitoring projects invite citizens to contribute data collection in different habitats and locations over a long period of time. Participants gain knowledge about the organisms they observe and are involved in the realisation of scientific research. Developing and implementing these projects to achieve scientific knowledge and scientific literacy requires great effort (Bonney et al. 2009); but citizen science represents a practical way to achieve the geographic scope required to document ecological patterns and address ecological questions at scales related to regional population trends and the effects of environmental processes such as the range and migration patterns of species (Sih 2013). Large-scale citizen science projects enable participants to join national and even global research and collect data in many places at the same time. The results of these studies can be used for population management decisions and even international environmental and conservation policies (Chandler et al. 2017a). Furthermore, the development of mobile applications for monitoring has brought together numerous new volunteers in nature conservation (Silvertown et al. 2013). Smartphone apps and mobile web access enable volunteers to be involved in recording observations and environmental monitoring in multiple ways (Luna et al. 2018). Also, the development of digital tools allows professionals to easily obtain large, comprehensive sets of data which would not be achievable without the contribution of volunteers.

Most biodiversity-oriented citizen science programmes aim to identify the location and abundance of species. These data are used in different studies (e.g. eBird, iNaturalist, and iSpot) to determine the population trends and range of species. For instance, more than 50% of GBIF (Global Biodiversity Information Facility) data for biodiversity monitoring is obtained from citizen scientists (Chandler et al. 2017b). The high participation rate is important in reducing data errors, as these projects generally do not require participants to have a scientific background. Other citizen science projects are carried out on specific topics by museums and local nature observation groups. *BioBlitzes* are popular in this regard and contribute to the confirmation of existing species, the discovery of new species, and knowledge about changes in the distribution of species, such as the expansion of invasive alien species (IAS) over time and space (Chandler et al. 2017b).

Considering the world is facing increasingly rapid and dramatic changes to habitats, species loss, and ecosystems due to human activity (UNGA 2015), there is an urgent need to monitor global biodiversity worldwide. Currently, there is great untapped potential for citizen science, particularly in Asia and Africa, to become a valuable tool for sustainable development (Pocock et al. 2019). Environmental citizen science is already widespread throughout North America, Australia, and Europe (Chandler et al. 2017b). For instance, the development of bird surveying in Turkey represents a valuable example for the application of citizen science in the life sciences by performing observations (Box 5.1).

### Box 5.1: eKuşBank and the Turkish Breeding Bird Atlas

Bird surveys carried out in Turkey from the nineteenth century until the 1970s were usually based on faunistic checklists and the addition of new records. The Ornithological Society of Turkey decided to compose a breeding bird atlas in the mid-1970s through new research approaches. However, this attempt was unsuccessful because not enough birdwatchers were available (Kasparek 1991). Between 2000 and 2002, Turkish bird atlas studies have been conducted on a regional scale with the support of the Society for the Protection of Nature (DHKD) and the methodology adopted by the European Bird Census Council (EBCC), and their breeding codes started to be used in Turkey for the first time.

With the foundation of *eKuşBank*, in 2004, a voluntary network has been established (Özesmi and Per 2006). Its breeding codes provide up-to-date, important data about the breeding behaviour of different species. They also increase the awareness of birdwatchers and scientists.

Since the breeding codes started to be used by the Ministry of Agriculture and Forestry in 2007 and their use in the ministry’s biodiversity projects was made obligatory (Nuhun Gemisi 2019), their usage has become widespread throughout the country. In addition, the data quality and the number of citizen observations are increasing (Per 2018). Since the completion of the breeding atlas project in 2019 (Boyla et al. 2019), areas that are significant in the breeding of species have come to light. *eKuşBank* data are widely and effectively used by scientists, NGOs, and decision-makers in Turkey. Following the inclusion of *eKuşBank* in the eBird infrastructure, now foreign tourists can also share their observations. Through citizen science, birdwatching has come to the fore in Turkey. The Department of Biology, Gazi University, in Ankara, is a project partner.

One of the few projects involving citizen scientists in doing experiments, that is, in contributing to testing hypotheses, is Heavy Metal City-Zen. Participants are asked to conduct a simple experiment in their urban gardens, by cultivating the same focal plant species in two different sorts of soil: the proposed variant (e.g. a mixture with compost that is provided to participants) and their own control variant (the untreated urban soil of their own garden). The project is still ongoing and aims to investigate the status of soil health in Vienna, Austria, providing data on the potential risks of heavy metal contaminants and suggesting mitigation strategies.

Similarly sparse are examples regarding the engagement of citizen scientists in theoretical projects, which are approaching and generating new research questions or discussing the relevance and potential of results. The few examples seem to focus on *crowdsourcing*, mainly used by companies which aim to include potential customers in production by conceiving new products, for instance, via the Samsung Strategy and Innovation Center – a global hub for start-ups, technology, and artificial intelligence professionals where new products are developed with input from the public.

### Scientific Achievements

The examples mentioned so far with respect to citizen science and the natural sciences share the common feature of citizen science being applied as a research method aiming for scientific outputs. Volunteers are engaged in the production of scientific knowledge, and their contributions are handled through scientific standards. Accordingly, citizen science projects in the natural sciences have led to peer-reviewed publications across a range of disciplines (Kullenberg and Kasperowski 2016).

Astronomy is one of the oldest scientific areas where lay people have contributed observations. It involves a broad array of persons, physically as well as via online platforms, and results in various scientific contributions: citizen scientists contribute to the detection of objects such as planets, comets, asteroids, and supernovae. They contribute to the understanding of the meteorology of planets by documenting clouds and storms; support insights into exoplanet systems and the radiation of blazar outbursts via observations; and cluster particles, craters, and supernovae based on digital images (Marshall et al. 2015). One of the biggest projects is Galaxy Zoo, part of the Zooniverse platform (Fortson et al. 2012): scientists outsourced the basic classification of galaxies. The project grew rapidly – gaining more participants and results than ever expected. In addition, the citizen scientists interacted, discussed results, and initiated their own project ideas, thereby increasing understanding about the scientific field. Zooniverse itself hosts different projects from a variety of disciplines where lay people can add their observations or contribute to digital projects to cluster patterns and observations. The contribution of citizen scientists is judged to be successful by the academic scientists involved; in one example, a Quasar ionization echo was discovered by a citizen scientist (Lintott et al. 2009).

In recent years, the amount and complexity of data from large detectors such as the Laser Interferometer Gravitational-Wave Observatory (LIGO) have grown enormously and exceeded the time capacities of volunteers. In a search for a new computational technology, researchers who established the Gravity Spy project tested a joint workflow between citizen scientists who identified novel glitches and machine learning techniques (Coughlin et al. 2019; cf. also Franzen et al., Chap. 10.1007/978-3-030-58278-4_9, this volume).

Additional applied research questions in the natural sciences have been successfully answered by citizen science. A Swedish group of scientists asked pupils to collect mushrooms and measure their radioactivity to investigate the long-term impact of the nuclear accident in Chernobyl (Andersson-Sundén et al. 2019). By joining the project, *Strålande Jord*, the pupils gain an understanding of the methodology of measuring radioactivity and additionally contribute to updating data on the radioactive load of mushrooms. Citizen scientists also contribute to the measurement of air pollution (i.e. aerosols). Within the framework of the iSpex experiment, participants contribute to the understanding of the temporal dynamics of aerosol distribution (Snik et al. 2014). Currently, iSpex is part of the MONOCLE (Multiscale Observation Networks for Optical monitoring of Coastal waters, Lakes and Estuaries) platform which develops low-cost optical sensors for citizen science.

In some other areas, for instance, chemistry, citizen science is largely absent. Albeit alchemists can be claimed as historical citizen scientists, nowadays there is scarce literature, and science and technology studies (STS) scholars complain that ‘fewer historians of technology focus on chemistry than on other sciences, for example, and virtually no social scientist covers mid- and late-20th-century chemistry’ (Woodhouse et al. 2002, pp. 305–306).

A further, broad field of natural sciences involving citizens is the life sciences, especially biology. As already mentioned in the introduction, for centuries humans observed natural phenomena and contributed to the understanding of nature. They described species and observe the spatial and temporal (phenological) distribution of plants and animals. Many projects today deal with the observation and interpretation of wildlife data, often with management approaches, supported by tools for data collection and analysis (Frigerio et al. 2018).

In contrast to the data that are observed and reported by citizen scientists from conservation areas and public land (e.g. backyards, gardens, and schools), biodiversity of agricultural areas is less well documented. In Germany, for instance, over 50% of the total land area is used for conventional and organic agriculture. Most of this land is designated private land, and comprehensive statements about the state of biodiversity in agricultural landscapes are limited. Therefore, nationwide monitoring schemes for agricultural areas are currently developed and tested to allow scientifically based answers about the influence of agricultural production, land use, and agricultural structural change on biodiversity. The Federal Ministry of Food and Agriculture in Germany is financing a 5-year pilot study to develop a basis for future monitoring of biodiversity in agricultural landscapes (MonViA). The aim of this pilot is the development of standardised sampling methods and analysis routines and the implementation of feasibility studies for different monitoring approaches, including a citizen science-based monitoring approach. Actors in the agricultural landscape hold extensive local knowledge about biological diversity, management practices, and the effects of the application of supplements such as fertilisers, some of which is maintained over generations. In turn, understanding the important role of biodiversity in the agricultural landscape and the added value of biodiversity for ecosystem stability varies considerably among actors. Further, the potential of citizen science in agricultural landscapes to contribute to reporting on Sustainable Development Goals (SDGs), such as ending hunger and achieving food security, has recently gained attention (Fritz et al. 2019). Established citizen science projects with and for farmers, such as the Austrian project *Biodiversitätsmonitoring mit LandwirtInnen*, show that citizen science can develop from an educational project to a monitoring programme. This project also highlights that participation, as well as non-participation, in citizen science should not incur negative economic or social consequences. Finally, citizen science in agricultural landscapes (as elsewhere) needs human power and financial capacity.

Participants in several international projects have contributed to the knowledge and conservation of genetic diversity, especially in the area of agriculture and food science (Ryan et al. 2018). More specifically, citizen scientists monitor pests, experiment with new food items, and assess the effects of environmental schemes, for example, flower stripes on their farms.

In genetics there is a vivid and diverse citizen science community analysing genomes, but also experimenting with new genetic sequences and synthetic organisms. One example is the experimental cultivation of *Roseobacter* strains, a common bacterium in oceans which may be used as chassis for synthetic biology applications, for instance, to degrade plastic. Using simplified devices, such as the UCLHack12 open-source incubator-shaker, investigations and manipulations are open to a broader public, in this case mainly cooperation between do-it-yourself (DIY) biologists and students (Borg et al. 2016). DIY biology comprises non-professional researchers (*biohackers*) who work in their own kitchen labs according to an ethical code of safety and transparency issues. In Europe, the community is challenged by strict legal regulations of gene technology and insufficient resources (Seyfried et al. 2014).

### Societal Outcomes

In general, applying citizen science in the natural sciences produces long-term societal outcomes. As participation is often not dependent on the competence or experience levels of volunteers, the research questions addressed do not usually include investigation of the short-term added value for participants (Kasperowski et al. 2017). There is empirical evidence that collaboration between education and natural science research increases motivation for out-of-school learning (Scheuch et al. 2018) and fosters the acquisition and retention of non-traditional knowledge compared to classroom-based curriculum learning (Hirschenhauser et al. 2019). Nevertheless, citizen science projects need adequate financial and temporal resources for recruiting (and often training) participants and communicating with them. The efficacy of citizen science is optimised when the tasks required can be learned quickly; and the impact of citizen science increases when citizen scientists feel responsible for and are personally involved in projects (Senabre et al., Chap. 10.1007/978-3-030-58278-4_11, this volume). Finally, the engagement of citizens in scientific processes has the potential to combine the collection of publishable data with outreach, thereby raising awareness and providing direct benefits to society without compromising scientific output.

## Challenges

As science professionalised, society and science diverged into strictly separated systems. The increasing specialisation and complexity of scientific language, research, and infrastructures, such as the publication system, make it challenging for lay people to access, gain, and benefit from knowledge that is ultimately generated by public finance. On the one hand, scientists conduct research in which millions of euros are invested, the legitimacy of which is sometimes questioned by the public. On the other hand, we are challenged by pressing problems in social, economic, and ecological contexts and demand sustainable solutions, which might be optimally fostered by science and society working together in a synergistic way. However, there are discipline-related differences, in the case of environmental issues and biodiversity problems, for instance, engaging and raising awareness by citizens can support science in bringing a topic to political interest, not least because the mass participation of citizens (and potential voters) indicates its importance. As Carl Friedrich von Weizsäcker observed, when the ‘socially organized search for knowledge’,^2^ science does not find its way into social and political space; novel forms of participation as well as new forms of teaching the natural sciences and access to scientific thinking are needed.

A key challenge is to understand and support participation in citizen science not only by citizen scientists but also by the initiators of citizen science (academics, data aggregators, policy-makers). Based on a theoretical framework on behavioural theory which differentiates between internal beliefs, social pressures, and control beliefs, Wehn and Almomani (2019) identified key incentives and barriers. For citizens, fun, interest, and recognition are supporting factors, while inadequate data use and neglect of privacy issues are hindering factors. For scientists, resources (time, staff, funding) play a key role alongside data quality (cf. Balint et al., Chap. 10.1007/978-3-030-58278-4_8, this volume). For both, management, data aggregation, and communication skills are also important. Furthermore, current science management approaches encourage short-term research projects with results applicable to decision-making processes rather than long-term commitments where the output/input ratio can be low. Good communication of the results and a well-defined data policy are important steps to enhance the impact of citizen science activities. Ganzevoort et al. (2017) report that only a minority share their data publicly and suggest viewing the citizen scientist as curator rather than as owner of the data as they care about how it is used. Here new concepts such as *dynamic informed consent* (Tauginienė et al., Chap. 10.1007/978-3-030-58278-4_20, this volume) may help.

## Relevance, Future Trends, and Recommendations

**Fig. 5.1:**
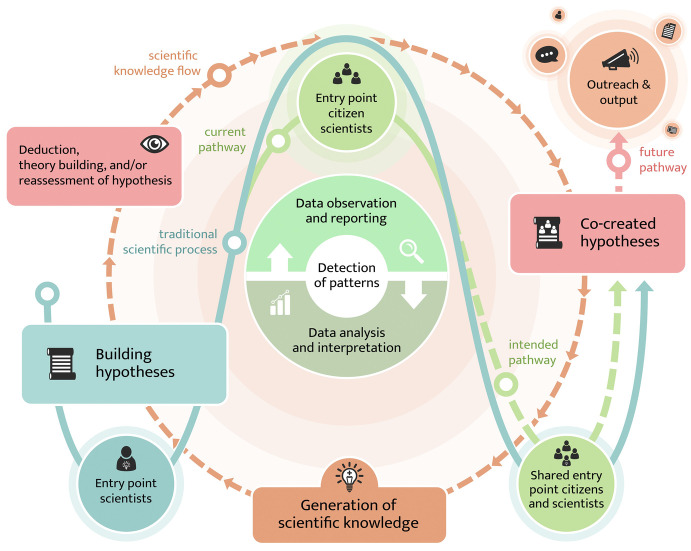
In the natural sciences, citizen scientists typically join the scientific process via crowdsourcing or data collection (solid green line). However, there would be added value if citizens were more integrated in the theoretical work/hypothesis-driven research (green dotted line)

Citizen science has been and will continue to be highly relevant to the natural sciences. Seeking understanding of the natural world is at the core of the natural sciences as well as being a goal for citizens. For this purpose, many hours of volunteering are spent to support the vast diversity of crowdsourced projects where citizens contribute by mass observations, while they are not necessarily deeply involved epistemically. From a data scientist perspective, crowdsourcing is the only way to gather comprehensive observation data for ensuring models’ accuracy. Watson and Floridi (2018) even argue that seldom or improbable events and anomalies, detected through the power of the crowd, are essential to further develop scientific theories. The integration of many (volunteer) observers increases the probability of detecting these seldom events. In addition, the ‘crowd’ can react quickly; for instance, the first projects to address the COVID-19 crisis emerged on the platform Foldit (McGrath 2020). In parallel, rather simple digital tasks are increasingly being replaced by machine learning and other automated systems. From a citizen’s perspective, crowdsourced projects are easy to join and do not often require much preparation, while comprehensive skills, advanced tools, and materials are not prerequisites for participation. A shortcoming of such low-level engagement is the missed opportunity for more advanced and in-depth involvement in scientific processes (Fig. 5.1) – engagement often ends with the data observation, recording, and transfer to platforms or the scientific community.

In fact, a transformation in respect to opportunities for citizens to engage in natural sciences beyond data contribution is already in sight. Natural science projects currently being classified as *activist approaches* may gain relevance in the future. For instance, citizens can search for scientific methods to demonstrate the severity and distribution of air pollution or participate in environmental justice (Toos et al., Chap. 10.1007/978-3-030-58278-4_19, this volume). Other activities engage citizens in the formulation of research questions (Senabre et al., Chap. 10.1007/978-3-030-58278-4_11, this volume). The added value of such approaches is multifaceted for both citizens and the scientific community. The expansion of opportunities for citizens to engage in various phases of the scientific discovery, such as engagement in theoretical work (Fig. 5.1), will likely increase citizens’ scientific literacy and understanding of the relevance and innovative power of the natural sciences in our daily and scientific lives. If planned carefully, the scientific community will see a growth of perspectives that will help to better illustrate a comprehensive view of the issues to be solved and the challenges to be addressed.

We expect that future citizen science in the natural sciences will maintain its focus on crowdsourcing activities, aiming to expand the spatial and temporal scope of traditional science. However, and this is our vision for future citizen science in the natural sciences, facilitating engagement of citizen scientists in all phases of the scientific process will contribute to a better understanding of the value of evidence-based decision-making (Herrick et al. 2018). Co-creation of knowledge with citizens has shown to have outcomes on multiple levels, particularly on the community and individual levels. Attainment of voice in decision-making, influence on management of natural resources, and the ability to access otherwise unavailable knowledge are only some of the outcomes driven by citizen science activities (Tero 2013; Bela et al. 2016). Based on the several examples mentioned as good practice in this chapter, we are confident that contemporary citizen science in the natural sciences has the untapped potential to contribute to the formulation of hypotheses and research questions. In other words, the future of citizen science in the natural sciences rests on the transition of citizen science beyond data collection (Fig. 5.1).

To enable multiple entry points to the scientific process for citizens, it is necessary to change the preconditions of the design and implementation of citizen science in the natural sciences. We recommend the following steps:

- First, establish and value flexible citizen science schemes that respond to the needs of volunteers to become more integrated participants in the whole scientific process.
- Second, provide on-going training for both volunteers and scientists, for example in scientific thinking to develop a scientifically literate mindset that leads to new research questions and theoretical thinking.
- Third, develop capacities for inter- and transdisciplinary research and communication and learn from best-practice case studies (e.g. on the platform EU-Citizen.Science; Butkevieciene et al., Chap. 10.1007/978-3-030-58278-4_16, this volume).
- Finally, value and formally acknowledge scientists who are open minded to this kind of trustworthy cooperation between scientists and citizens.

Literally shifting frontiers was part of the motivation of the past explorers of the natural world. Without confidence to sail beyond the horizon, without curiosity to enter the unknown wilderness, and without true passion for the natural world, much of our current knowledge would be fragmented and less colourful. It is now up to us to prepare and enthuse the next generation of Humboldts, Müllers, and Merians. Citizen science holds many opportunities to contribute to the natural sciences and to experience the beauty of understanding the world around us.

## Footnotes

1 https://www.youtube.com/watch?v=cE1kpXLkGbo

2 Cited in the discussion forum: Die Verantwortung der Wissenschaft in der Gesellschaft. Öffentliche Diskussionsveranstaltung am 03. Mai 2017 in der Aula der Georg-August-Universität Göttingen. https://vdw-ev.de/portfolio/die-verantwortung-der-wissenschaft-in-der-gesellschaft/
